# Thromboelastometry in veal calves to detect hemostatic variations caused by low doses of dexamethasone treatment

**DOI:** 10.1186/1746-6148-9-55

**Published:** 2013-03-26

**Authors:** Antonio Borrelli, Claudio Bellino, Elena Bozzetta, Barbara Bruno, Sara Falco, Cristiana Maurella, Paola Gianella, Marzia Pezzolato, Aurelio Cagnasso, Antonio D’Angelo

**Affiliations:** 1 University of Turin, Grugliasco (TO), 10095, Italy; 2 Istituto Zooprofilattico Sperimentale del Piemonte, Liguria e Valle D’Aosta, Turin, 10154, Italy

**Keywords:** Veal calves, Dexamethasone, Illicit treatment, Thromboelastometry, Hypercoagulability

## Abstract

**Background:**

The illegal administration of hormones, steroids, β-agonists and other anabolic agents to productive livestock in the European Union continues, despite the long-term ban on their use and despite the measures provided under the directives to monitor certain substances and residues thereof in the interest of protecting consumer health and animal wellbeing. Often administered in low doses in the form of a drug cocktail, these compounds escape detection by common analytical techniques. The aim of this study was to determine whether low-dose dexamethasone administration (0.4 mg orally per day, for 20 days) in white-meat calves produced variations in blood coagulation, as measured by thromboelastometry. A second aim was to determine whether such variations could be valid in detecting illicit low-dose dexamethasone treatment.

**Results:**

The study population was 42 Friesian calves kept under controlled conditions until 6 months of age. The calves were subdivided into 2 groups: a control group (group A, n = 28) and a group treated with dexamethasone (group B, n = 14) for 20 days beginning at 5 months of age. When compared against the age-matched control group, the dexamethasone-treated calves showed a significant increase in alpha angle, maximum clot firmness and a significant decrease in clot formation time on all thromboelastometric profiles (P < 0.05). The clotting time was significantly decreased on the in-TEM® profile but increased on the ex-TEM® and fib-TEM® profiles (P <0.05). The Receiver Operating Characteristic curves, plotted for the Maximum Clot Elasticity (MCE), had a cut-off value ≥488.23 mm for in-TEM® MCE [Se 85.7%, (95% CI 57.2-98.2); Sp 100% 96.43% (95% CI 81.7-99.9] and a cut-off value ≥63.94 mm for fib-TEM® MCE [Se 92.8 (95% CI 66.1-99.8); Sp 89.3% (95% CI 71.8-97.7)]. In order to increase the sensitivity of the test two parameters (in-TEM® and fib-TEM® MCE) were used as two parallel tests; subsequently, the sensitivity rose to a point value of 99% (95% CI 85.4-99.9).

**Conclusions:**

Thromboelastometry identified a state of hypercoagulability in the dexamethasone-treated subjects. Furthemore, the results of this preliminary study suggest that TEM may be useful in the detection of illicit low-dose dexamethasone treatment.

## Background

Synthetic corticosteroids and their residues constitute a potential risk for human health, as they can mimic, block or interfere with hormone actions [1]. For therapeutic indications only, the use of some glucocorticosteroids is allowed and therefore maximum residue limits have been established for bovine edible tissues (0.75 μg per kg in kidney and muscle, and 2 μg per kg in liver) and milk (0.3 μg per kg).^a^

The illegal administration of hormones, steroids, β-agonists and other anabolic agents to productive livestock in the European Union continues, despite the long-term ban (Council Directives 96/22 EC and 96/23 EC) on their use and despite the measures provided under the directives to monitor certain substances and residues thereof in the interest of protecting consumer health and animal wellbeing. Numbering among the most widely used illicit anabolic agents are β-agonists (clenbuterol and ractopamine), sex hormones (estradiol, testosterone and nortestosterone) and corticosteroids (dexamethasone and betamethasone) [2,3]. Often administered in low doses in the form of a drug cocktail, these compounds escape detection by common analytical techniques such as chromatograph/mass spectrometry and liquid chromatography tandem mass spectrometry which provide unreliable results. For years the scientific community has worked to identify possible markers for illicit drug treatments and to find which markers would be useful in additions to traditional detection methods. Screening methods to investigate either the temporary or the permanent effects of the use of such drugs could be useful and some of them have already been put in place [4-7]. On the contrary there are still some effects that have to be further investigated, such those on hormonal, hematological and biochemical parameters.

In vitro and in vivo studies on the effects of corticosteroids on blood coagulation in humans have identified increased clotting factors (e.g., factor VIII or von Willebrand factor) and decreased fibrinolysis [8-11] which could lead to hypercoagulability. Furthermore, thrombotic and thromboembolic events have been reported as a complication of hyperadrenocorticism in both human and canine patients [9,12,13]. In Cushing’s syndrome, or in exogenous steroid administration, alterations of the hemostatic system have been described in human beings and in dogs [9-11,14-16].

Presently, there is no gold standard for the diagnosis of hypercoagulability; however, one of the most reliable methods for coagulation monitoring and testing is the viscoelastic technique applied in both human medicine [17-19] and veterinary medicine [20-26]. Invented by Hartet in 1948, the technique involves whole blood assessment that takes into account the plasma and the cellular components of hemostasis. Standard coagulation profiles end as soon as fibrin strands appear, whereas viscoelastic technique continues through the following steps of clot development [27]. Moreover, it allows the assessment of the hypercoagulable state, which is very difficult to evaluate with routinely used coagulation assays.

Three instruments are currently used in medicine: Sonoclot; Thromboelastography (TEG); and Thromboelastometry (TEM).

The aim of this preliminary study was to determine whether TEM could detect variations in coagulation induced by low-dose dexamethasone administration (0.4 mg orally per day, for 20 days) in white-meat calves. A second aim was to determine whether such changes as measured by TEM could identify illicit dexamethasone treatment.

## Methods

This study is an integral part of a research project approved by the Ministry of Health and the Bioethics Commission. The study was conducted according to animal welfare considerations and regulations, and the protocol was approved by the Italian Ministry of Health (RF-IZP-2006-364645).

### Animals

A total of 122 male Friesian veal calves (age range, 15–35 days) were bought from local breeders, randomly divided into two groups matched for body weight and age, and farmed in two boxes under the same conditions for 6 months [6]. Each box had its own crib, multiple drinking troughs, and a dedicated automated milk feeder system. To protect the animals against infections, all were vaccinated against infectious bovine rhinotracheitis, parainfluenza-3 virus, bovine respiratory syncytial virus and bovine viral diarrhea virus. Clinical controls were carried out daily by a veterinarian, and the animals entering the study had received no medical treatment during their lifespan. During the sixth month, 81 animals received dexamethasone 21-(disodium phosphate) (0.4 mg per day) orally for 20 days and the remaining 41 animals were used as controls. Further details on the trial design can be read in Bozzetta et al., 2011.

Among the largest population that entered in the trial, two subgroups were randomly extracted: a control group (group A, n = 28) and a dexamethasone-treated group (group B, n = 14). These two subgroups entered this study. The drug was administered orally for 20 days (0.4 mg per day) beginning at 5 months of age. Upon completion of treatment (day 20), blood from both groups was drawn for sampling via jugular venipuncture using minimum stasis and avoiding vessel damage. When samples were difficult to obtain, they were discarded and blood collection was repeated from the contralateral jugular vein. The blood was collected with 21G needles and divided into 2 test tubes, one containing Na-citrate (Venosafe 3.8%, Terumo; for a final ratio of 9:1) for analysis of hemostasis, and the other containing K3-EDTA for Complete Blood Count (CBC).

### Hemostasis

Coagulation was analyzed via rotational thromboelastometry (ROTEM®, TEM Innovation GmbH). TEM was evaluated directly in the field within 30 minutes of blood collection, as directed by the manufacturer’s instructions, and the analyses were run for 30 minutes. Three profiles (in-TEM®, ex-TEM®, and fib-TEM®) were differentiated with the addition of specific reagents: the in-TEM®, containing ellagic acid, is used to study the intrinsic pathway; the ex-TEM® reagent, containing tissue factor (thromboplastin, factor III), is used to study extrinsic coagulation; and fib-TEM®, containing cytochalasin D, an inhibitor of platelet function, allows qualitative evaluation (fibrin polymerization) of fibrinogen.

**Figure 1:**
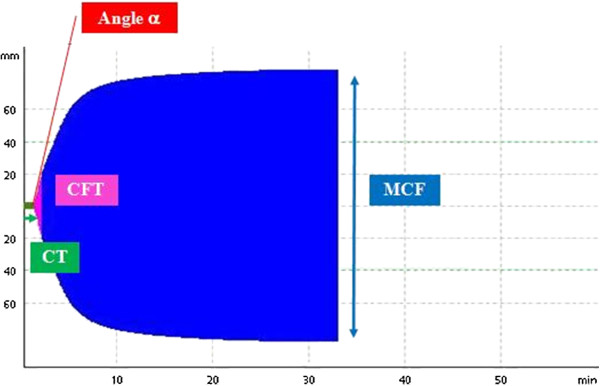
**Example of a normal thromboelastogram (ex-TEM^® ^profile).** CT: Clotting time; CFT: Clot formation time; MCF: Maximum clot firmness.

Thromboelastometry provides a reaction curve (Figure 1) representing various different physiological events mediated by the interaction of platelets, coagulation factors, fibrinogen and the fibrinolysis system. The numerical parameters are derived from a detailed mathematical analysis of the reaction curve. The first parameter obtained is the clotting time ([CT], s), which is mainly affected by the concentration of plasma coagulation factors and coagulation inhibitors (e.g., antithrombin or drugs) [27]. This parameter is related, but not identical, to traditional coagulation tests (prothrombin time and activated partial thromboplastin time). The clot formation time ([CFT], s) and α angle (α, degree), which is the slope of the tangent to the curve, express the velocity of clot formation and are affected predominantly by platelet number and function, as well as by fibrinogen activity. Maximum clot firmness ([MCF], mm) is determined by both platelet and fibrin formation in the presence of factor XIII [27]. Moreover, as it seems that MCF does not reflect the actual physical properties of cloth strength according to Hooke’s law, a new parameter was calculated by transforming MCF: maximum clot elasticity (MCE), given by (MCFx100)/(100-MCF), as described in Lang et al., 2009 [28].

### CBC tests

CBC were performed (ADVIA® 120 Hematology System, Siemens Diagnostics) simultaneously with cytological analysis of a fresh blood smear.

### Statistical analysis

The thromboelastometry parameters were expressed as the median, minimum and maximum value. Reference values obtained from the control group were expressed as 5th – 95th percentile. The differences between the control and the treatment groups were evaluated using the Wilcoxon rank sum test. A value of P < 0.05 was considered significant. To identify a cut-off value that could correctly separate the maximum number of treated animals from the controls, a Receiver Operating Characteristic (ROC) curve was plotted for the MCE parameters.

On the basis of the results given by ROC curves, the best discriminatory value was used to calculate the validity of the MCE parameters in correctly identifying treated and control animals.

Furthermore the results of the assessment of the parameters (i.e. sensitivity of MCE) were used to evaluate the sample size useful to detect the absence of illicit treatment in a bovine farm. A sample size for freedom from treatment was calculated by assuming the low band of the Confidential Interval (CI) of the sensitivity (Se) of the MCE parameters (i.e. worst case scenario), a finite population (100–500 animals), a confidence level of 95%, and a design prevalence of 33%. To improve the diagnostic sensitivity, two parameters (fib-TEM MCE and in-TEM MCE) were joined and used as two parallel tests [29].

Statistical analysis was performed using Software Stata 11 MP and sample size was calculated by using the EpiTools1, a web-based suite of epidemiological. ^b^

## Results

**Figure 2:**
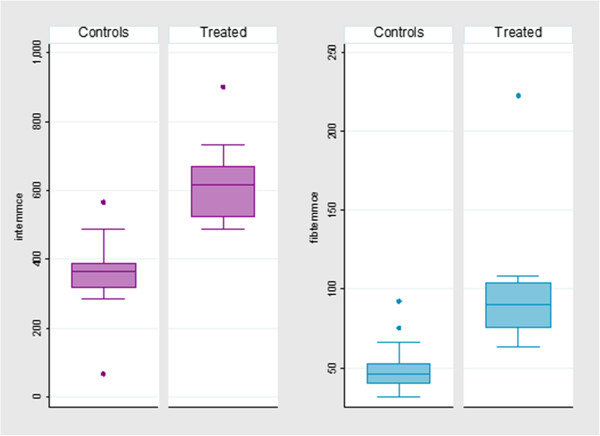
**Box-plots representing the significant difference in in-TEM^® ^and fib-TEM^® ^MCE values between the animals treated with corticosteroids and the control ones (P < 0.05).**

Table 1 reports the TEM results for group A and group B and the statistical comparison between the two groups. When compared against the age-matched control group, the treatment group showed a significant increase in alpha angle and MCF, and a significant decrease in CFT on all profiles. The CT was significantly decreased on the in-TEM® profile but increased on the ex-TEM® and fib-TEM® profiles. As representative of the difference between the two groups, boxplots of in-TEM® MCE and of fib-TEM® MCE are shown in Figure 2.

**Table 1:** Results of thromboelastometry in control calves (group A) and dexamethasone-treated calves (group B)

	**CT sec**		**CFT sec**		**MCF mm**		**ALPHA degree**	
	**Group A**	**Group B**	**Group A**	**Group B**	**Group A**	**Group B**	**Group A**	**Group B**
in-TEM	257	221.5*	59.5	36.5*	78.5	86*	78	83*
	172.8-360.7	169.6-275	42.4-79.3	29-46.4	74.4-82.7	83-88.7	75-82	80.7-84
ex-TEM	74	84*	94	57.5*	78.5	86*	75	78*
	58.8-88.2	69.9-105.7	67.1-118.7	50-79.8	72.4-84.7	78.3-89.4	70-77.7	75.7-80
fib-TEM	74.5	82.5*	104	59.5*	31.5	47.5*	76.5	78*
	61.1-83	71.2-97.8	68.1-322.1	47.3-83.8	25.4-42	41-58	71.7-79	75-80.3

* Statistically significant differences between the control and the treated group (P < 0.05).

(Values are expressed as median and 5th-95th percentile).

*CT*: Clotting Time; *CFT*: Clot Formation Time; *MCF*: Maximum Clot Firmness.

**Figure 3:**
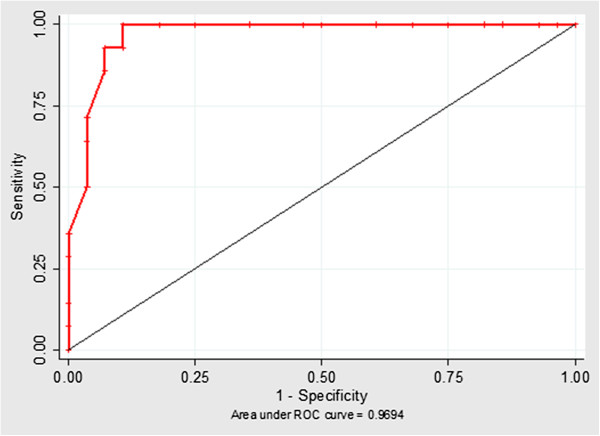
**ROC curve of the fib-TEM^® ^maximum clot elasticity (MCE).** MCE parameter shows a good accuracy in discriminating treated and controls.

**Figure 4:**
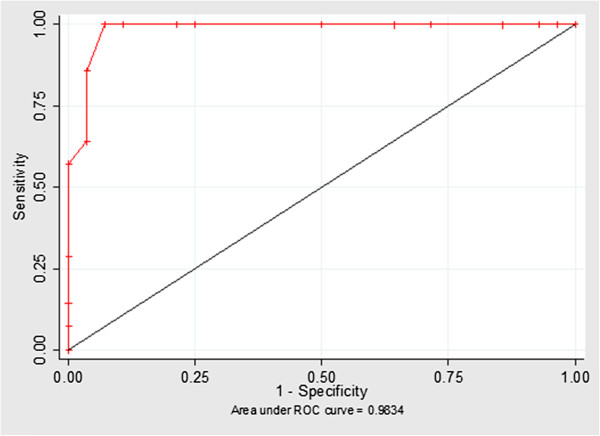
**ROC curve of the in-TEM^® ^maximum clot elasticity (MCE).** MCE parameter shows a good accuracy in discriminating treated and controls.

The ROC curves indicating the best discriminatory power between the treated and control groups had a cut-off value ≥488.23 for in-TEM® MCE [Se 85.7%, (95% CI 57.2-98.2); Sp 96.43% (95% CI 81.7-99.9] (Figure 3) and a cut-off value ≥ 63.94 for fib-TEM® MCE [Se 92.8 (95% CI 66.1-99.8); Sp 89.3% (95% CI 71.8-97.7)] (Figure 4). In order to increase the sensitivity of the test two thromboelastometric parameters (in-TEM® MCE and fib-TEM® MCE) were used as two parallel tests; subsequently, the sensitivity rose to a point value of 99% (95% CI 85.4-99.9). In this way, the two variables might be used together as a unique test.

Calculation of the sample size useful to exclude the presence of any treatment in a herd size of a finite population showed that: 1) in the first situation, taking into account each MCE parameter one at time and setting a 95% confidence level, a design prevalence of 33% (equal to the prevalence of treated of this study), and the level of sensitivity given by the low band of the CI (i.e. 57.2%) the sample size would be 16 heads when using the in-TEM MCE; and 14 heads when using the low band of the CI of the fib-TEM® MCE (i.e. 66.1%) 2) under the same assumptions and with the low band sensitivity obtained in the parallel tests (Se = 85.4%), the sample size was 11 heads.

CBC revealed a hypochromic microcytic anemia in both groups. All subjects presented hemoglobin levels >4.5 mmol per L, as required by European regulations (Directive 97/2/EC and Italian Decree Law 331/98) that determine the minimum standards for the protection of calves.

## Discussion

In veterinary medicine, TEM/TEG has been used to investigate different conditions of hypocoagulabilty [25,30], and hypercoagulabilty [21,23,31] in dogs and, to a lesser extent, in other species [20,24]. In bovine medicine, only one preliminary study on thromboelastometry [32] has been published to date. In this study the analytical performance of the instrument in the bovine species has been evaluated and coefficients of variation have been calculated. In general, in cattle, as in dogs [33], the use of strong clotting activators (as the in-TEM® and ex-TEM® reagents), instead of recalcification only (as in na-TEM®) reduce the impact of pre-analytical phactors on TEM tracings and allow more reproducible results. Furthermore it is not known at this moment if also in cattle, as in human beings, the TEM results are influenced by age and gender (Sucker et al. 2011). For this reason further studies are necessary to investigate the impact of age, gender and also breed category (e.g. dairy vs. beef breed) on TEM results.

The results of the present study indicate a hypercoagulable condition in the dexamethasone-treated group (increase in alpha angle and MCF with a reduction in CFT on all profiles; decrease in CT on the in-TEM® profile), with the involvement of both plasma and cellular components of hemostasis. Interestingly, the CT was shortened on the in-TEM® profile of the treated calves but prolonged on the ex-TEM® and fib-TEM® profiles. This difference could be explained by an increase in the activity of factor VIII and XI, as observed in humans after corticosteroid administration [10]. Both factors are involved in the intrinsic pathway (in-TEM®), whereas the profiles triggered by tissue factor (ex-TEM® and fib-TEM®) are less influenced by these changes. The reason for CT prolongation in the ex-TEM® and fib-TEM® profiles is not clear. Further qualitative assessments of the hemostatic process (e.g., plasma factor levels) are needed to elucidate these changes.

In human studies, hypercoagulability after corticosteroid administration has been related also to an increase in fibrinogen levels [10]. This condition could be the cause for the increase in the MCF observed in this study. A significant increase in MCF was observed not only on the in-TEM® and ex-TEM® profiles but also on the fib-TEM® profile, where platelet contribution to clot stability is annulled by the addition of cytochalasin D. Here, again, further studies are needed to quantify the contribution of factor XIII. A limitation of this study is the lack of fibrinogen concentration measurement.

Certain preanalytical factors are known to influence the thromboelastometric results in different species (e.g., sampling technique and storage condition) [24,33]. Also, because TEG tracings can be altered in some pathologic conditions due to an increase or a decrease hemostasis [21,23,34,35], it is essential to standardize sampling technique and storage conditions and to evaluate the patient’s clinical status when performing TEM analysis, as was done in the present study.

The results of the ROC curves show that parameters such as in-TEM MCE and fib-TEM MCE are able to identify cattle treated with dexamethasone for long periods at low doses. These preliminary results could be useful in field identification of illicitly treated cattle, even in a fairly small (n = 11) sample of heads simultaneously treated in each herd.

All the calves in both groups were affected by anemia due to the particular zootechnic. This variation in hematocrit is known to affect the TEM tracing, also in bovine species, since it increases the blood coagulability measured by the instrument [32]. It is not known whether this phenomenon reflects an in vivo effect of red blood cell mass on hemostasis or whether it is simply an artifact of the viscoelastic technology [27]. In our study, anemia was not considered as a confounding factor, because both groups showed the same severity of anemia. It is not known, at this moment, if the anemia observed in our study population, could have enhanced the hemostatic changes secondary to dexamethasone treatment. For this reason, further studies are necessary to investigate the effects of dexamethasone in bovines not affected by anemia.

To the best of authors’ knowledge this is the first study to assess the effect of steroids on blood coagulation in bovines.

## Conclusions

The results of this preliminary study suggest that TEM may be useful in the detection of illicit low-dose dexamethasone treatment. Further studies on larger populations are needed to improve the accuracy of results. While TEM holds promise as a tool for use in the field, other relevant data are necessary in order to apply thrombolestometry in the detection of illicit drug treatment: the method’s analytical sensibility threshold in relation to various dosages, the treatment duration, and the length of time following treatment during which altered hemostasis can still be reliably detected.

## Endnotes

^a^Commission Regulation (EC) No 508/1999 of 4 March 1999 amending Annexes I to IV to Council Regulation (EEC) No 2377/90 laying down a Community procedure for the establishment of maximum residue limits of veterinary medicinal products in foodstuffs of animal origin. Official Journal of the European Community, L60, 16–52.

^b^http://epitools.ausvet.com.au.

## Abbreviations

CFT: Clot formation time; CT: Clotting time; CBC: Complete blood count; CI: Confidential interval; MCE: Maximum clot elasticity; MCF: Maximum clot firmness; ROC: Receiver operating characteristic; Se: Sensibility; Sp: Specificity; TEG: Thromboelastography; TEM: Thromboelastometry

## Competing interests

All authors declare that they have no competing interests.

## Authors’ contributions

Conception and design: AB, AD. Acquisition and interpretation of the data: AB, BB, SF, CM. Collection and assembly of data: AB,CB, BB, SF, AD. Provision of study materials or patients: AB, CB, AC, MP, EB, AD. Statistical expertise: CM. Drafting the article: AB, BB, SF, PG, AD. Critical revision of the article for important intellectual content: All authors. All authors read and approved the final manuscript. Acquisition of funding: MP, EB, AD.
